# Lithopedion in a Geriatric Patient

**DOI:** 10.1055/s-0038-1676038

**Published:** 2018-12-12

**Authors:** Andrés Ricaurte Sossa, Henry Bolaños, Andrés Ricaurte Fajardo, Ángela Camila Burgos, Valentina Garcia, Paola Muñoz, Diego Rosselli

**Affiliations:** 1 Department of Obstetrics and Gynecology, Hospital Universitario Departamental de Nariño, San Juan de Pasto, Colombia; 2 Medical School, Pontificia Universidad Javeriana, Bogota, Colombia; 3 Medical School, Universidad Cooperativa de Colombia, San Juan de Pasto, Colombia; 4 Department of Surgery, Hospital Universitario Departamental de Nariño, San Juan de Pasto, Colombia

**Keywords:** lithopedion, ectopic pregnancy, geriatric

## Abstract

Lithopedion (*lithos* = rock and *paidion* = child) is a rare condition that only occurs in 1.5 to 1.8% of extrauterine pregnancies and in 0.00045% of all pregnancies. It consists of an ectopic pregnancy in which the fetus dies but cannot be reabsorbed by the mother's body, which then coats it in a calcium-rich substance. We present the case of a 77-year-old woman with an incidental diagnosis of a lithopedion, which had been retained in her left pelvis for presumably 40 years.

## Introduction

Abdominal ectopic pregnancy, defined as implantation of the fertilized ovum in the peritoneal cavity, excluding tubal, ovarian or intraligamentary implantations, is an uncommon type of extrauterine pregnancy. It has an estimated incidence of 1 per 10,000 to 25,000 live births and leads to high maternal and fetal mortality.^1^ ^2^ Most of these pregnancies occur after tubal rupture, with a subsequent reimplantation in the peritoneal cavity. However, it can also occur that the zygote passes through the fallopian tube and is primarily implanted in the peritoneal cavity.^3^ ^4^ ^5^ Extrauterine pregnancies are sometimes not identified and may resolve spontaneously, even when gestation is advanced.

An extrauterine pregnancy that has calcified over time is known as lithopedion.^6^ The estimated incidence of lithopedion is 1.5 to 1.8% of extrauterine pregnancies. It usually occurs when a fertilized ovum attaches outside the uterus and the fetus starts to grow but cannot survive and dies.^7^ ^8^ If the dead fetus is too large to be reabsorbed by the mother's body, it is recognized as a foreign object by the mother's immune system, which reacts by coating the fetus in a calcium-rich substance that will eventually mummify and petrify the fetus body.^8^ ^9^

In the medical literature, there are ∼ 300 reported cases of lithopedion.^6^ ^7^ Lithopaedion was first described during the 10th century by an Arab physician. However, the most famous case was described by Jean d'Ailleboust, in 1582 in *The Lithopaedion of Sens*, which describes a female lithopedion retained for 28 years, discovered during the necropsy of a 68-year-old woman.^10^

Kuchenmeister^11^ describes three types of lithopedion according to the calcified structures. The first one, litokeliposis, presents calcification of the membranes without the calcification of the fetal body. The second one, litokelitopedion, is the calcification of the membranes and the fetus. The third one is true lithopedion, in which the fetus is infiltrated with calcium salts, but the calcification of the membranes is negligible.^7^ ^8^

The duration of lithopedion retention has been described to be between 4 and 60 years. For lithopedion development, the fetus has to remain alive for more than 12 weeks. Additionally, the ectopic pregnancy has to escape medical detection, and the fetus has to remain in aseptic conditions and in a favorable environment for calcification.^7^ ^12^ Detection can be difficult, and most cases are found incidentally during surgery, radiographic images or autopsy. It can be suspected in patients with persistent or recurrent abdominal pain, chronic constipation, intestinal obstruction or obstructive uropathy.^9^ ^13^

Computed tomography (CT), magnetic resonance imaging and barium enema have been described to be performed based on the patient's symptoms and are useful to plan the surgical approach. The treatment of these patients should be individualized, considering maternal age, presentation and symptoms. Main complications include intestinal obstruction, pelvic abscess, cephalopelvic disproportion in future pregnancies, extrusion of fetal parts through the abdominal wall, rectum and vagina; fistula formation and tubal infertility.^10^ ^13^ ^14^ ^15^ ^16^

We present the case of a 77-year-old patient with an abdominal lithopedion suspected to be 40 years old, incidentally discovered.

## Case Description

**Fig. 1:**
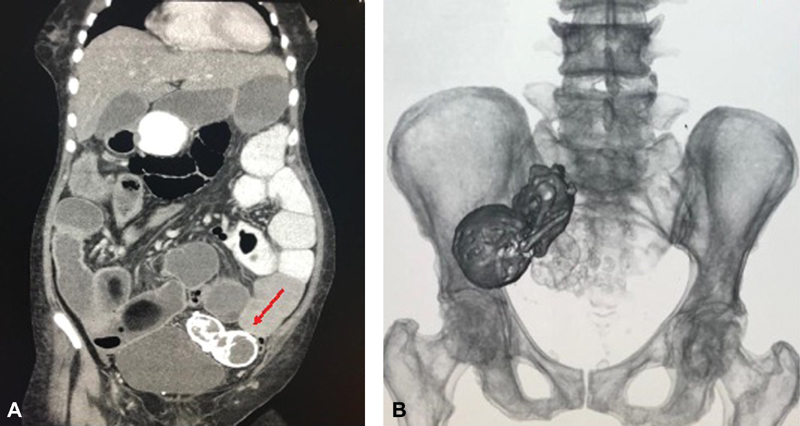
(**A**) Abdominal computed tomography evidencing the mummified fetus (**B**) Computed tomography imaging of the abdomen with 3D reconstruction.

**Fig. 2:**
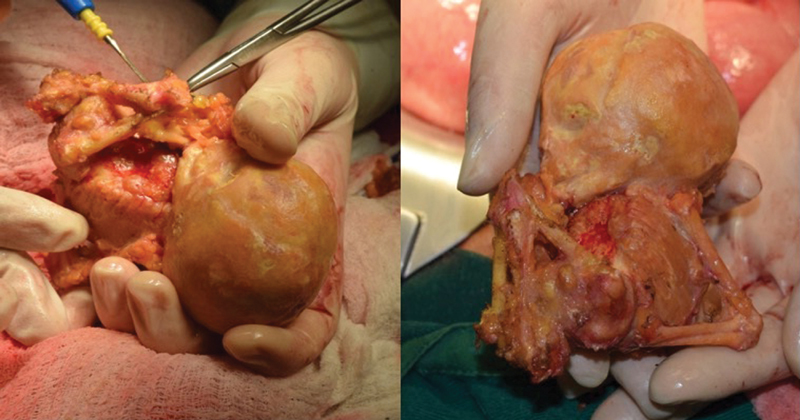
Mummified fetus extracted during laparotomy.

A 77-year-old female patient from Tumaco, in the Pacific coast of Colombia, presented with 8 days of generalized abdominal pain associated with symptoms of absent peristalsis. She referred 4 pregnancies, of which she had had 3 normal vaginal deliveries and 1 miscarriage (40 years prior), which was recognized by the patient as her last pregnancy. Additionally, she mentioned a family history of colon cancer. Upon admission to the emergency department, she was hemodynamically stable, with no signs of peritoneal irritation but a positive ascitic wave. There were no palpable masses, and symptomatic treatment was initiated. Blood tests were requested, which were not suggestive of intrabdominal infection. Plain abdominal X-rays did not show any significant finding; abdominal ultrasound documented a narrow hepatic angle and thickness in the right colon mucosa. Given that imaging findings were not conclusive, a contrasted abdominal CT was performed, revealing thickening of the ascending colon walls, signs of ileocolic intussusception and heterogeneous calcifications in the lower left pelvis compatible with a mummified fetus (Fig. 1). After these findings, a diagnostic laparoscopy was performed with no relevant findings. Therefore, laparotomy was performed finding a mummified fetus with 41 mm biparietal diameter (BPD), adhered to the greater omentum, which was dissected and extracted in block (Fig. 2). Subsequently, a right hemicolectomy was performed. She was then transferred to the intensive care unit (ICU), where she required ventilatory and vasopressor support. After two days in the ICU and a favorable evolution, she was transferred to the general ward and after resolution of the symptoms, she was discharged with no further complication.

## Discussion

Lithopedion is a rare entity with a variable clinical course. It may be asymptomatic for many years or, less frequently, it may present with persistent or recurrent abdominal pain, chronic constipation, intestinal obstruction or obstructive uropathy.^9^ ^13^ It can also present with complications such as pelvic abscess; cephalopelvic disproportion in future pregnancies^16^; extrusion of fetal parts through the abdominal wall, rectum or vagina; fistula formation or tubal infertility.^10^ ^13^

With the advances in antenatal diagnostic techniques of different fetal conditions, lithopedion is much less frequent. However, in neglected regions with difficult access to basic health services, as in the case presented, the timely identification of infrequent antenatal conditions might be difficult.^14^ Since the literature on this subject is mostly based on case reports, it is not clear what might be the best diagnostic tools or the most appropriate therapeutic approach. However, surgery has been the selected treatment option in most of the reported cases.

In this case, a lithopedion suspected to be 40 years old was found by means of a contrasted abdominal CT scan. In case it is not possible to correctly measure the length of the long bones, which is usually presented as anthropometric data, BPD is accepted as the most reliable measurement. In this case, it corresponded to a gestational age of at least 18 weeks.^15^ The intestinal obstruction that led to the request for intraabdominal images and, therefore, to the diagnosis of lithopedion had no relation to the intraabdominal fetus. In its absence, the diagnosis could have remained unnoticed.
